# Computational Design of Novel Hydrogen-Doped, Oxygen-Deficient Monoclinic Zirconia with Excellent Optical Absorption and Electronic Properties

**DOI:** 10.1038/s41598-019-46778-5

**Published:** 2019-07-15

**Authors:** Sarah A. Tolba, Nageh K. Allam

**Affiliations:** Energy Materials Laboratory (EML), School of Sciences and Engineering, The American University in Cairo, New Cairo, 11835 Egypt

**Keywords:** Electronic properties and materials, Photocatalysis

## Abstract

Monoclinic ZrO_2_ has recently emerged as a new highly efficient material for the photovoltaic and photocatalytic applications. Herein, first-principles calculations were carried out to understand how Hydrogen doping can affect the electronic structure and optical properties of the material. The effects of Hydrogen interstitial and substitutional doping at different sites and concentrations in m-ZrO_2_ were examined by an extensive model study to predict the best structure with the optimal properties for use in solar energy conversion devices. Hydrogen interstitials (Hi) in pristine m-ZrO_2_ were found to lower the formation energy but without useful effects on the electronic or optical properties. Hydrogen mono- and co-occupying oxygen vacancy (Ov) were also investigated. At low concentration of Hydrogen mono-occupying oxygen vacancy (HOv), Hydrogen atoms introduced shallow states below the conduction band minimum (CBM) and increase the dielectric constant, which could be very useful for gate dielectric application. The number and position of such defect states strongly depend on the doping sites and concentration. At high oxygen vacancy concentration, the modeled HOv-Ov structure shows the formation of shallow and localized states that are only 1.1 eV below the CBM with significantly high dielectric constant and extended optical absorption to the infrared region. This strong absorption with the high permittivity and low exciton binding energies make the material an ideal candidate for use in solar energy harvesting devices. Finally, the band edge positions of pristine and doped structures with respect to the redox potentials of water splitting indicated that Hydrogen occupying oxygen vacancies can increase the photocatalytic activity of the material for hydrogen generation due the extremely improved optical absorption and the band gap states.

## Introduction

Zirconia (ZrO_2_) is one of the most important wide band-gap transition metal oxides. It exists in three polymorphs. The monoclinic polymorph is the low-temperature phase (stable up to 1480 K), which is transformed into the tetragonal phase, and finally to the cubic phase as temperature increases^1^. Besides its large band gap, Zirconia exhibits some important properties, such as high strength, high fracture toughness and hardness, chemical inertness, and resistance to corrosion^2^. Moreover, it is one of the promising materials to replace silicon dioxide as a gate dielectric material in metal-oxide-semiconductor (MOS) transistors due to its high dielectric constant, relatively high band offsets with respect to silicon, and the good thermodynamic stability^3^. However, monoclinic Zirconia suffers from fixed charge and carrier traps due mainly to the presence of oxygen vacancy centers^4^. Those oxygen vacancies made Zirconia a good material for use in oxygen gas sensors^5^ and solid oxide fuel cells (SOFCs)^6^.

Therefore, many theoretical studies have been devoted to investigating the structural and electronic properties of pure and defected ZrO_2_. Ricca *et al*.^2^ studied the structure, the relative stability, and the electronic properties of the bulk and surface of the three ZrO_2_ polymorphs. They compared different methods and basis sets to identify the role of the exchange-correlation functional to determine the best parameters that yield reliable and accurate data. Zheng *et al*.^7^ studied the native point defects in ZrO_2_ as a function of the external chemical potential. Also, Foster *et al*.^8^ studied oxygen-related native defects formation energies and their energy levels, however the results suffered high uncertainties in defect formation energies and transition levels due to the band gap underestimation. To solve the bandgap underestimation problem, different hybrid density functional theory (DFT) functionals have been used. Van de Walle *et al*.^9^ calculated the formation energies and transition levels of native defects in ZrO_2_ using the Heyd–Scuseria–Ernzerhof (HSE) hybrid functional. Using the nonlocal B3LYP hybrid functional, Shi *et al*.^10^ studied the formation and migration of native defects in monoclinic Zirconia. Lyons *et al*.^4^ studied the stability of Hydrogen impurities, oxygen vacancies and oxygen interstitial defects along with their role as carrier traps and sources in monoclinic ZrO_2_. Although the use of hybrid functionals yields results that are close enough to the experimental results, they are computationally expensive. In this regard, Li *et al*.^11^ used Hubbard correction (DFT + U) approach as an alternative to accurately investigate the electronic and optical properties of monoclinic ZrO_2_ with a good agreement with the experimental findings while avoiding the excessive cost of the hybrid functional calculations. Generally, applying the Hubbard correction to wide band gap materials with 3d or 4d orbitals was found to accurately determine the materials properties^12^. Thus, DFT calculations can be used to accurately predict the properties of Zirconia.

Despite these exceptional properties of ZrO_2_, it is rarely being investigated for solar energy applications, mainly due to its wide bandgap. Recently, Sinhamahapatra *et al*.^13^ developed a black monoclinic ZrO_2−x_ with an exceptional increase in visible light absorbance due to the high density of oxygen vacancies that exhibited significant activity for solar light-induced H_2_ generation from methanol/water mixture with outstanding stability. As the case with black anatase (TiO_2−x_) and other metal oxides, the introduction of defects such as oxygen vacancies can improve the optical properties by introducing new states in the band gap, which can narrow the band gap and consequently increase sunlight harvesting^14^. However, besides the strong visible-light absorption, decreasing the recombination rate of charge carriers is needed to achieve high photocatalytic activity. Usually, flat and half-field deep localized bandgap states act as recombination centers of light-generated electrons and holes in bulk materials. To this end, Wang *et al*.^15^ introduced H-doped black anatase (TiO_2−x_H_x_) and found that hydrogenation of TiO_2_ tends to produce strong band tailing near the conduction band minimum, resulting in bandgap narrowing with better solar absorption (≈83%), significant low recombination, and consequently excellent photocatalytic activity. Since then, numerous experimental and theoretical investigations of the hydrogen doped rutile and anatase phases of TiO_2_ were performed. Soliman *et al*.^16^ studied the limitations of the hydrogenation process of TiO_2_ nanostructures and the relationship between the rutile/anatase ratio and the nature of defect states that control the nature and concentration of defect states. From the theoretical point of view, the nature of the created defect states due to the hydrogen doping and the relationship between the states and the position of the hydrogen atom inside the lattice of TiO_2_ were well-investigated^17–21^. Lately, Yang *et al*.^22^ reported a strong visible-light absorption red anatase as a result of the excitation of a new sub-valence band associated with two hydrogen filled oxygen vacancies through combined experimental and theoretical work. However, to the best of our knowledge, the effects and the models of hydrogen defects in pristine and Oxygen-deficient monoclinic zirconia for solar energy applications is yet to be investigated.

Therefore, the aim of the current work was to investigate, for the first time, the possible scenarios for doping monoclinic zirconia with Hydrogen and to elucidate the effects on light harvesting and photocatalytic activity of the material. First-principles calculations were performed to explore the effect of hydrogen doping and hydrogen-mediated oxygen vacancy in monoclinic ZrO_2_ at high and low concentrations and the controlling variables for defect formation. The effect of defects on the structural, electronic, and optical properties of the material was investigated. The article is organized as follows: (i) screening different doping strategies to identify the most stable and favorable defect structures, where the structural properties, formation energy, and thermodynamic stability were calculated, (ii) the bandgap, density of states, charge populations, and bond ionicity were investigated to study the atomic and the electronic structure of defected structures, (iii) the dielectric function and optical absorption were calculated to study the optical properties, and (iv) Finally, the band edge positions with respect to the redox potentials of water were calculated.

## Computational Methods

For all calculations, the spin-polarized density functional theory (DFT) calculations based on the generalized gradient approximation (GGA) with the exchange and correlation (PBE)^23^ as implemented in CASTEP code with the plane wave pseudopotential method^24^ were used. The interaction between the ionic core and valence electrons is represented via the Ultrasoft-pseudopotential^25^ and the electron wave function is expanded in plane waves up to a cutoff energy of 380 eV. For the irreducible Brillouin zone, the Monkhorst–Pack scheme k-points grid sampling was set with a spacing of grid points smaller than 0.05 with grid 4 × 4 × 4 for all geometry optimization calculations, and 0.04 spacing with grid 5 × 5 × 5 for density of states calculations^26^. Because of the self-interaction error in DFT^27^, the Hubbard U correction approach^28^ is used to accurately describe the energy band gap^29^ and consequently the defect formation energy and its states in the band gap^12^. The Hubbard U parameter was set at 4 eV for Zr 4d states. Also, the Hubbard correction was applied to the 2p states of oxygen as well to avoid the influence over the Zr–O covalent bond that happens when applying the correction only to the d states, thus it will shift while the 2p states of oxygen will not change^18^. Before the calculations of the electronic structures and optical properties, geometry optimization calculations of the structures of pure and defected m-ZrO_2_ were performed to identify the lowest energy structures. The position of all atoms is fully relaxed until the mean Hellmann–Feynman force acting on the atoms was less than 0.01 eV Å^−1^. The energy change, maximum stress, and maximum displacement tolerances in the optimization were set at 5.0 × 10^−6^ eV/atom, 0.02 GPa, and 5.0 × 10^−4^, respectively. To check the accuracy of our calculations, we compared the optimized pristine m-ZrO_2_ to the experimentally and theoretically reported structures. There are many different experimental reports for the monoclinic ZrO_2_ crystal structure but for our initial structure, we used the lattice parameters and the atoms’ coordinates from Purohit *et al*. work^30^. Using our computational setup, for the pure bulk m-ZrO_2_, the calculated lattice parameters and band gap are obtained as a = 5.24 Å, b = 5.2 Å, c = 5.4 Å, and E_g_ = 5.14 eV, respectively. These values are in good agreement with the previous experimentally and theoretically reported values^31^. Thus, the same setup has been used to optimize the defected structures.

**Figure 1:**
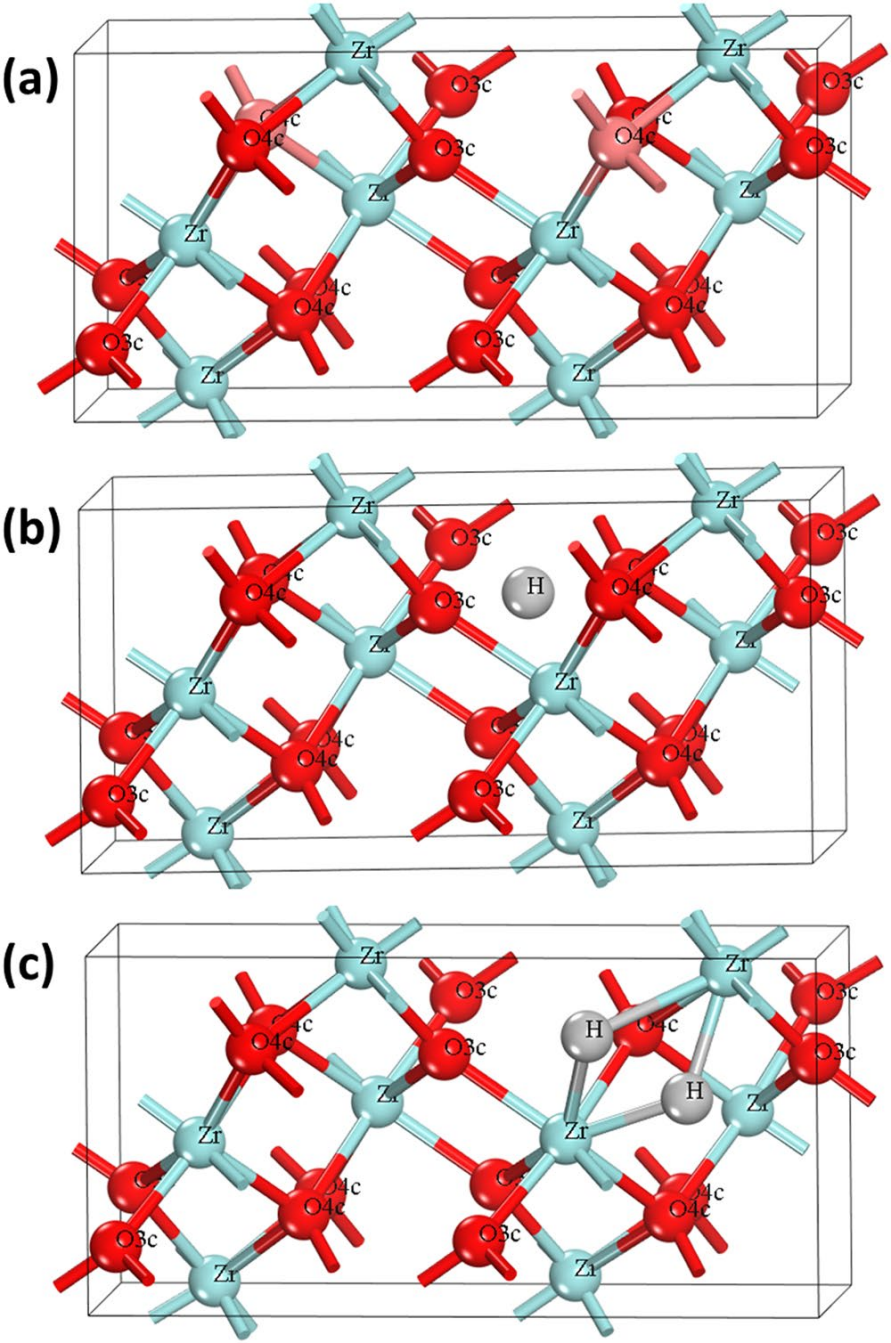
Initial crystal structure of (**a**) m-ZrO_2−x_ with the removed oxygen atoms colored in light red. (**b**) H_i_ and (**c**) (2H)_Ov_.

For the defected structures, a 2 1 1 supercell with total number of 24 atoms with defect concentrations of 0.125 and/or 0.25 nX/nZr (X = defect) was created by introducing one and/or two defect species, respectively. First, we simulated 0.125 and 0.25 oxygen vacancy defects in 2 1 1 supercell following the previous work by Sinhamahapatra *et al*.^13^ and compared with low and high concentrations see Fig. 1a. Then, we introduced hydrogen atom to perfect and reduced ZrO_2_ structures in both interstitial and substitutional (for oxygen) sites. First, with the pristine ZrO_2_, the interstitial and substitutional defects were examined. (i) For the substitutional position, since the monoclinic phase has two types of oxygen atoms, 4-coordinated (O_4c_) and 3-coordinated (O_3c_), and they are not equivalent, they would have different binding energies. Thus, two different models have been used where one H atom replacing oxygen atom in O_4c_ an O_3c_ positions (named H_o4_ and H_o3_, respectively). (ii) To simulate the Hydrogen in interstitial position, the initial structure was set as shown in Fig. 1b, where one and two hydrogen atoms (named H_i_) were set in almost equal distance to O_3f_ and O_4f_ atoms, followed by relaxation till reaching the lowest energy configuration. For the reduced ZrO_2_, it is more complex, and many defected configurations were simulated to study all possible structures, depending on the various preparation techniques for introducing hydrogen to oxygen-deficient structures. Starting with 0.125 and 0.25 4-coordinated oxygen vacancy concentrations corresponding to one and two oxygen vacancies in the cell (named 1O_v_ and 2O_v_, respectively), the simulated configurations are as fellow: (i) Single hydrogen atom sitting in oxygen vacancy of one oxygen-vacancy structure (named 1H_Ov_). (ii) As the hydrogen amount increases, we simulated two hydrogen atoms in oxygen vacancy of one oxygen-vacancy structure (named (2H)_Ov_). In the structure with two oxygen, (iii) one hydrogen atom in one oxygen vacancy while the other vacancy still empty (named H_Ov_-O_v_) that with low hydrogen concentration. By increasing the amount of Hydrogen, we studied the possibility of (iv) the second hydrogen atom sitting in the unoccupied vacancy (named 2H_Ov_), or (v) the second hydrogen atom will prefer to co-occupy the occupied vacancy with hydrogen atom before occupying all empty vacancies first (named (2H)_Ov_-O_v_). The simulated configurations were built to cover all the possible hydrogen defective structures and the best structure for targeted applications.

## Results and Discussion

The predicted change in the electronic and optical properties of zirconium oxide and oxygen-deficient zirconium oxide caused by hydrogen incorporation in both the interstitial and substitutional positions are investigated.

### Defect formation energy and structures

To investigate the relative difficulty for defect incorporation and the relative stability of the doped structures, the defect formation energy was calculated. As the formation energy of point defect strongly depends on the chemical potentials, the defect formation energy (E_*form*_) was calculated as:

1 $${E}_{form}={E}_{total}(final)-{E}_{total}(initial)-nx\mu {X}_{added}+ny\mu {Y}_{removed}$$

where E_total_ (final/initial) is the total energy of the final and initial structures, nx and ny are the numbers of added and removed elements, respectively, and μX_added_ and μY_removed_ are the chemical potentials of the added and removed atoms that depend on the experimental growth conditions, which can be Zr-rich or O-rich for the extreme ones. Under the O-rich condition, μ_O_ can be calculated from the ground state energy of O_2_ molecule, while μ_Zr_ and μ_H_ can be obtained as μ_Zr_ + 2μ_O = _μ_ZrO2_ and 2μ_H_ + μ_O_ = μ_H2O_, respectively. Under the Zr-rich (O-poor) condition, μ_Zr_ is the energy of one Zr atom in bulk Zr and μ_H_ can be calculated from the ground state energy of H_2_ molecule. The μ_O_ can be obtained via the growth conditions, where μ_Zr_ + 2μ_O_ = μ_ZrO2_. The formation energies of all studied defects are listed in Table 1. For detailed investigation, we studied the structural distortion caused by the defects via the crystallite volume and lattice parameters of pure and doped m-ZrO_2_ as listed in Table 2. Structural distortion modifies the electronic environment and as a result should affect the photocatalytic properties.

**Table 1:** Formation energy (E_*form*_) of all defected monoclinic ZrO_2_ structures.

Structure	Formation Energy (E_*form*_), eV	
	O-rich	Zr-rich
1Ov	5.973	3.042
2Ov	11.963	6.1
Ho_3_	7.496	2.9
Ho_4_	7.839	3.244
H_i_	3.845	2.181
2H_i_	6.73	3.402
Ho_v_	1.866	0.202
(2H)_Ov_	3.219	−0.101
Ho_v_-O_v_	1.842	0.178
(2H)o_v_-O_v_	3.132	−0.136
2Ho_v_	2.362	−0.606

**Table 2:** Lattice parameters of pure and possible defected structures of monoclinic ZrO_2_.

Structure	Lattice Parameter (Å)			Volume (Å)^3^
	a	b	c	
ZrO_2_	10.49	5.22	5.41	292.0
1Ov	10.49	5.21	5.41	291.8
2Ov	10.5	5.2	5.41	291.4
H_i_	10.59	5.23	5.44	296.2
2H_i_	10.8	5.28	5.43	303.6
H_Ov_	10.5	5.22	5.43	293.6
(2H)_Ov_	10.54	5.19	5.45	294.0
H_Ov_-Ov	10.51	5.22	5.43	293.5
2H_Ov_	10.55	5.19	5.51	297.8

First, for oxygen vacancies, based on Sinhamahapatra *et al*.^13^, O_4f_ vacancy is more energetically favorable than the O_3f_ vacancy counterpart. Thus, in our oxygen-deficient structures, one and two neutral O_4f_ atoms were removed to simulate different concentrations. With oxygen vacancy in the cell, the adjacent Zr atoms to the vacancy have shorter bonds with the other oxygen atoms by 0.01 A and the cell ended up with smaller volumes by 0.215 and 0.576 for single and double vacancy, respectively, Table 2. From Table 1, 0.125 concentration of oxygen vacancy is much more favorable and less energetic than the 0.25 concentration. Regarding the hydrogen doping, we first simulated hydrogen doping in pristine ZrO_2_ with 0.125 concentration in substitutional (for O atom) and interstitial positions. Then, the possible doping structures at higher concentration (0.25) were also investigated. The hydrogen atom in interstitial position prefers bonding to one O3c instead of O4c atom. Much less energy is required to get hydrogen atom in interstitial position than in the substitutional position because of the unneeded Zr-O bond breaking that requires additional energy. To increase H_i_ concentration, extra 1.22 eV is needed. Starting with one O_v_ in the cell, the structure with two H atoms co-occupying the vacancy ((2H)_Ov_) is more favorable (in Zr-rich condition) than the one H atom in the vacancy (H_Ov_) structure, where H atoms will get the electrons that remain in the cell after vacancy formation. To get more insights into the (2H)_Ov_ structure, two configurations based on two hypotheses were simulated. Do the H atoms prefer to first co-occupy the vacancy or to mono-occupying all vacancies before co-occupying? To answer this question, we used a structure with two oxygen vacancies, a configuration where 2H atoms co-occupying one vacancy ((2H)_Ov_-Ov) and the other configuration H atoms mono-occupying the vacancies (2H_Ov_) to represent the two hypotheses, respectively. The results showed the H atoms to mono-fill all the vacancies first before the co-filling. Thus, co-occupying will only happen if the Hydrogen concentration is higher than the concentration vacancies in the material.

These results guided us to the last configuration with two vacancies in a cell where one of them is filled with one hydrogen atom while the other is empty (H_Ov_-Ov), representing a lower hydrogen concentration than the vacancy concentration in the material. However, whether 2H_Ov_ is more or less favorable than H_Ov_-O_v_ depends on the fabrication conditions and doping strategy. From structural point of view, while oxygen-vacancy decreases the volume, hydrogen doping either in interstitial or oxygen position increases the volume of the cell. The largest volume is for 2H_i_, and the smallest for both is found with 2O_v_.

**Figure 2:**
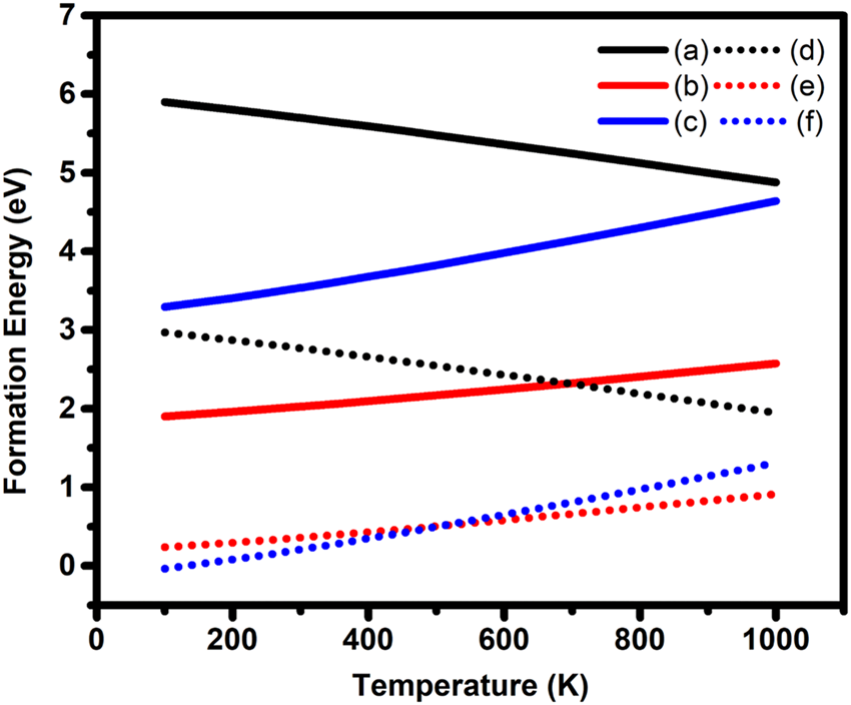
Temperature-dependent formation energy of (**a**) 1Ov, (**b**) H_Ov_, (**c**) (2H)_Ov_ in oxygen rich conditions, (**d**) 1Ov, (**e**) H_Ov_, and (**f**) (2H)_Ov_ in Zirconium rich conditions.

Furthermore, the complexity of forming oxygen vacancy and hydrogen occupied oxygen vacancy was studied based on the diversity of the fabrication temperature. Using the dependence of the gas chemical potential on the temperature^32^, the defect formation energy was calculated at different temperatures^33^, Fig. 2. Generally, the formation energy of oxygen vacancy decreases with increasing the temperature, while the energy for mono-or co-occupying the vacancy increases. At temperatures higher than 600, the H_Ov_ is more thermodynamically stable than the (2H)_Ov_ and thus the formation of the (2H)_Ov_ can be prevented.

### Electronic structure

**Figure 3:**
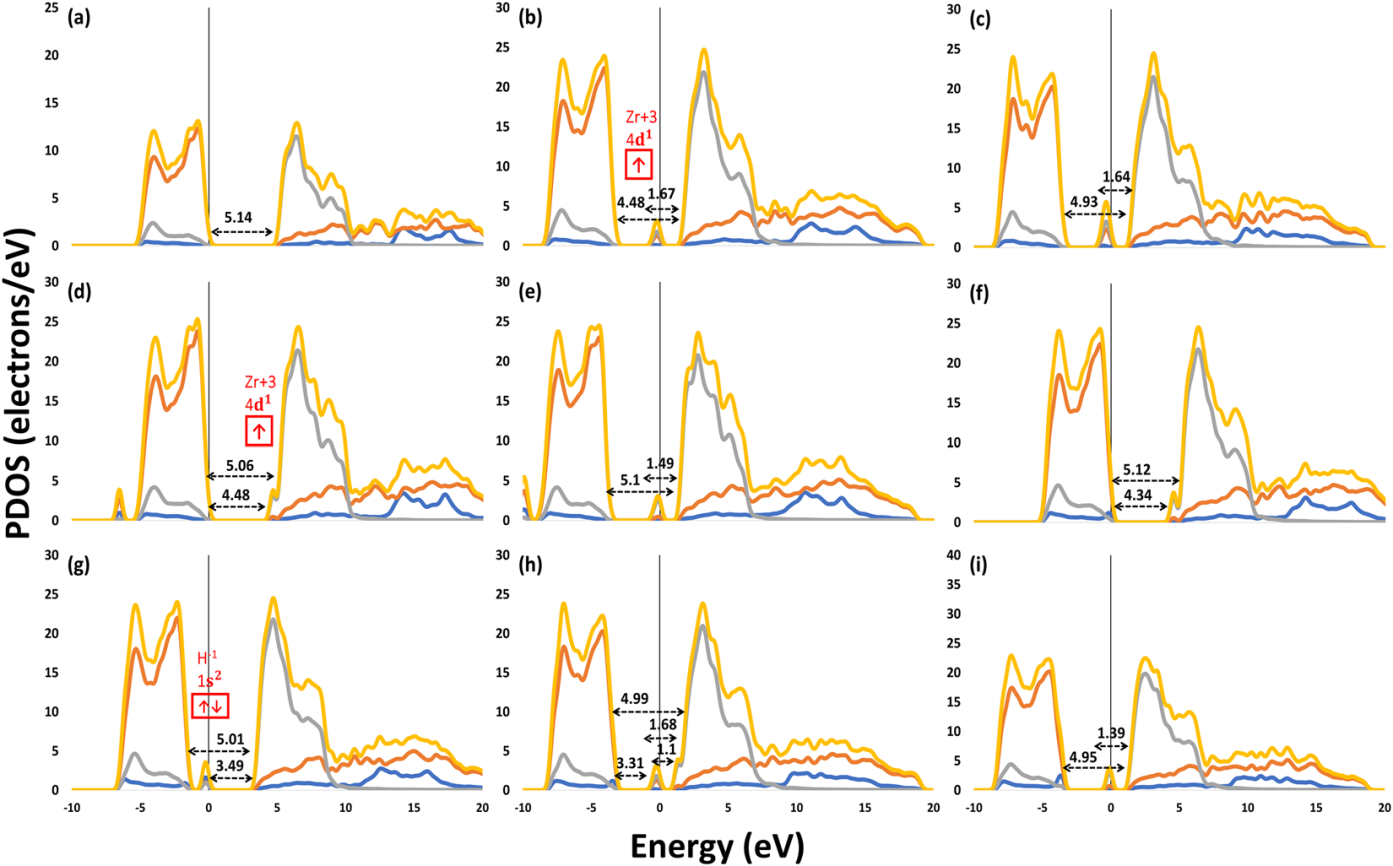
PDOS of (**a**) perfect ZrO_2_, (**b**) 1V_O_, (**c**) 2V_O_, (**d**) 1H_i_, (**e**) 2H_i_, (**f**) 1H_ov_, (**g**) (2H)ov, (**h**) (H)_ov_-O_v_, and (**i**) 2H_ov_.

To get a deeper insight into the contribution of atomic orbitals in the electronic structure of the materials, the bandgap narrowing, defect states, and the electron partial density of states (PDOS) along with the charge populations of perfect and defected m-ZrO_2_ structures and the population ionicity index (Pi) were calculated as illustrated in Fig. 3 and Table 3. The bond population ionicity was calculated using Eq. 2. Note that 0 < Pi < 1 represent the pure covalent and pure ionic type, respectively. Pc is the bond population for a purely covalent bond, thus Pc = 1, and P is the bond population as in Mulliken charge population.

2 $${P}_{i}=1-{e}^{-|\frac{Pc-P}{P}|}$$

**Table 3:** Atomic and bond Mulliken charge population of the studied compositions.

Structure	Bonds	Atoms	Mulliken Charge	Bond Population	Ionicity index (Pi)
ZrO_2_	(Zr-O_3c_)	Zr	+1.69	0.44, 0.39	0.72, 0.79
	(Zr-O_4c_)	O_3c_	−0.83	0.3, 0.28	0.9, 0.92
		O_4c_	−0.86		
O_v_	Zr-O_v_-Zr	Zr	+1.4, +1.46	—	
H_i_	Zr-(O_3c_-H_i_)	Zr	+1.52, +1.6	0.7	0.35
		Hi	+0.33		
H_Ov_	(Zr-H_Ov_-Zr)	Zr	+1.47, +1.52	0.25, 0.13	0.95, 0.99
		HO_v_	−0.44	0.05, −0.01	
(2H)_Ov_	(Zr_1_-H_1_-Zr_2_)	Zr_1_	+1.52	0.18–0.2	0.99, 0.98
	(Zr_1_-H_2_-Zr_2_)	Zr_2_	+1.55	0.22–0.26	0.97, 0.94
		H_1_	−0.41		
		H_2_	−0.28		

From bond populations and ionicity index (Pi) of pure ZrO_2_, the O_3c_ bond to Zr atom is more covalent than the O_4c_-Zr bond. Thus, the O_3c_-Zr bond is stronger, which agrees with the findings that O_4c_ vacancy is easier to form. Oxygen vacancies introduce a localized Zr^3+^ states in the bandgap, Fig. 3b,c. From Mulliken charge, this state consists of 0.29 and 0.23 electron charges added to the Zr atoms. Increasing the vacancy concentration resulted in narrowing the bandgap and shifting the Zr^3+^ state closer to the conduction band minimum (CBM). The new band gap in the 2O_v_ structure was found to be 1.64 in good agreement with previous experimental and theoretical reports^13^.

Hydrogen defects in the interstitial or substitutional O_v_ positions introduce tail (shallow) defect states bellow the CBM, Fig. 3d,f, which become localized in the bandgap when the concertation increases, Fig. 3e,i. Hydrogen as a cation with +0.33 charge in the H_i_ structure has a strong covalent bond to the O_3c_ atom with 0.7 bond population. However, as an anion with −0.44 charge in the H_Ov_ structure, it bonds to only two Zr atoms with an ionic bond. The H-Zr bond states (1 s^2^) are deep in the valance band in the cases of mono-filling the O_v_ while it becomes localized above the valance band maximum in the co-occupying structure ((2H)_Ov_) and leads to the formation of a new sub-valance band as can be seen in Fig. 3f,i,j. From H_Ov_ PDOS, the localized state of the vacancy band becomes partly delocalized as a shallow energy level near the conduction band minimum and is shifted more bellow the CBM and becomes localized when the concentration increases as in the case of 2H_Ov_ structure. This is in an excellent agreement with Lyons *et al*. using the same high concentration of Hydrogen defects, indicating that H_Ov_ introduces localized state and acts as a fixed charge^4^. However, there is a disagreement with their findings at the low concentration, which drives us to the assumption that decreasing the O_v_ concentration before introducing H can solve the fixed charge and the problems of trapping of charge carriers. In the H_Ov_-O_v_ structure, both O_v_ and H_Ov_ states are presented as a localized state at 1.1 eV and a shallow state below the CBM, resulting in a decrease in the recombination of charge carriers and a remarkable bandgap narrowing, leading to an excellent optical absorption and favorable CBM position. Overall, the PDOS of all defected structures show that all states are well close to either the VBM or the CBM, which is an advantage as the localized states in the middle of the band gap act as recombination centers. Finally, despite the flaw of Mulliken charge population method, as the other various methods used to assign the electronic population and charges in computational techniques, it is obviously seen that it can accurately describe the charge population with good agreement with the PDOS results.

### Optical properties

**Figure 4:**
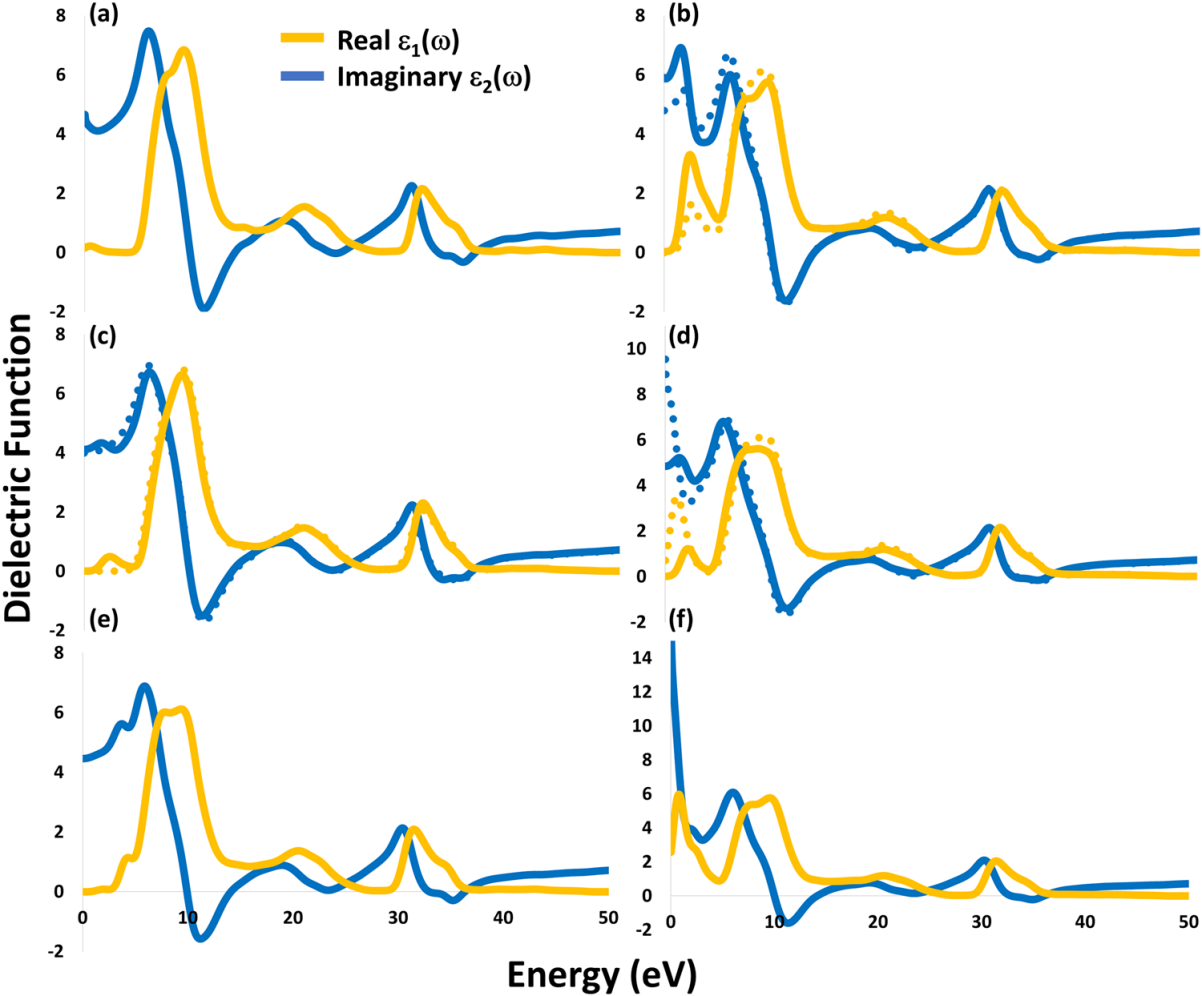
Dielectric function of (**a**) Perfect ZrO_2_, (**b**) O_v_, (**c**) H_i_, (**d**) H_ov_, (**e**) (2H)_ov_, and (**f**) H_ov_-O_v_. Solid and dashed lines represent 0.25 and 0.125 concentrations, respectively.

The complex dielectric function and the optical absorption spectrum were calculated to investigate the optical properties of the materials^34^. The effect of the defect type and concentration on the dielectric constant, from the real part of dielectric function at Zero electron volt, was studied. Hence, the material with higher dielectric constant indicates to higher permittivity, lower exciton binding energy, and lower recombination rate, which should improve the charge carrier extraction efficiency upon their use in energy conversion devices. Comparing the dielectric constant of pristine ZrO_2_ (4.66) and defected-structures, increasing O_v_ concentration increases the dielectric constant from 4.79 to 5.91 while increasing H_Ov_ concentration decreases it from 9.54 to 4.85 and H_i_ concentration showed no effect on the dielectric constant (4.11). The highest dielectric constant was found for the H_Ov_-O_v_ structure with a dramatic increase to 17.36, see Fig. 4.

**Figure 5:**
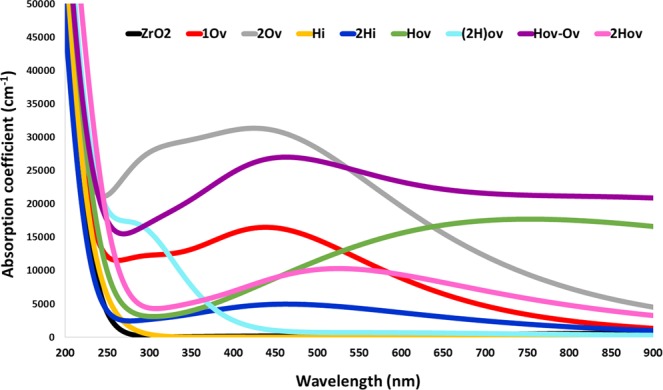
The optical absorption coefficient of pure and defected m-ZrO_2_.

From the main peaks of the imaginary part, the electronic transition from the valence band (occupied orbitals) to the conduction band (empty orbitals) can be studied. Thus, the peaks give an insight into the electronic transition from O 2p or H 1 s of the valence band to the Zr 4d or H 1 s of the conduction band, in addition to the electron(s) transition between the partly/fully occupied orbital close to the Fermi level into the un-occupied orbital, which can be used to explain the optical absorption of the studied structures. Also, a higher imaginary part of the dielectric function at zero electron volts refers to stronger interactions between electrons and photons, which leads to better absorption light characteristics. From Figs 4 and 5, the following findings can be realized: (i) Oxygen vacancy in 1O_v_ and 2O_v_ structures strongly increases the absorption compared to that of pure ZrO_2_. The imaginary part of the dielectric function has a peak at ~2.8 eV and its strength increase from 1.5 to 3.1 upon increasing the concentration, indicating more electronic transition from VB to CB. However, at zero electron volts, the imaginary part equals zero, which indicates lower interactions between electrons and photons compared to 0.16 in the pristine ZrO_2_. The absorption also increases when the concentration of oxygen vacancy increases and starts to decrease from 525 nm in both concentrations. (ii) The H_i_ structure has almost no enhancement in absorption that can be explained by the absence of the peaks in the dielectric function, which indicates no transition of electrons from VB to CM. However, at higher H_i_ concentration (2H_i_ structure), a small peak is realized with slight absorption increase. (iii) With H_Ov_ structure, the imaginary part at zero eV is shifted to 0.7, which refers to an increase in the interaction between electrons and photons and explains the absorption increase. At higher concentration (2 H_Ov_ structure), a significance increase in the VB to CB electronic transition with a decrease in electron-photon interaction and absorption was observed. (iv) In (2H)_Ov_ structure, a deep small peak is observed with a remarkable increase in the optical absorption in the UV region, which could be useful for transparent solar cells. (v) Finally, the highest absorption all-over the spectrum along with the highest electrons and photons interaction, where the Imaginary part = 2.58 at zero eV, was observed for the H_ov_-O_v_ structure.

### Band structure and band edge positions

**Figure 6:**
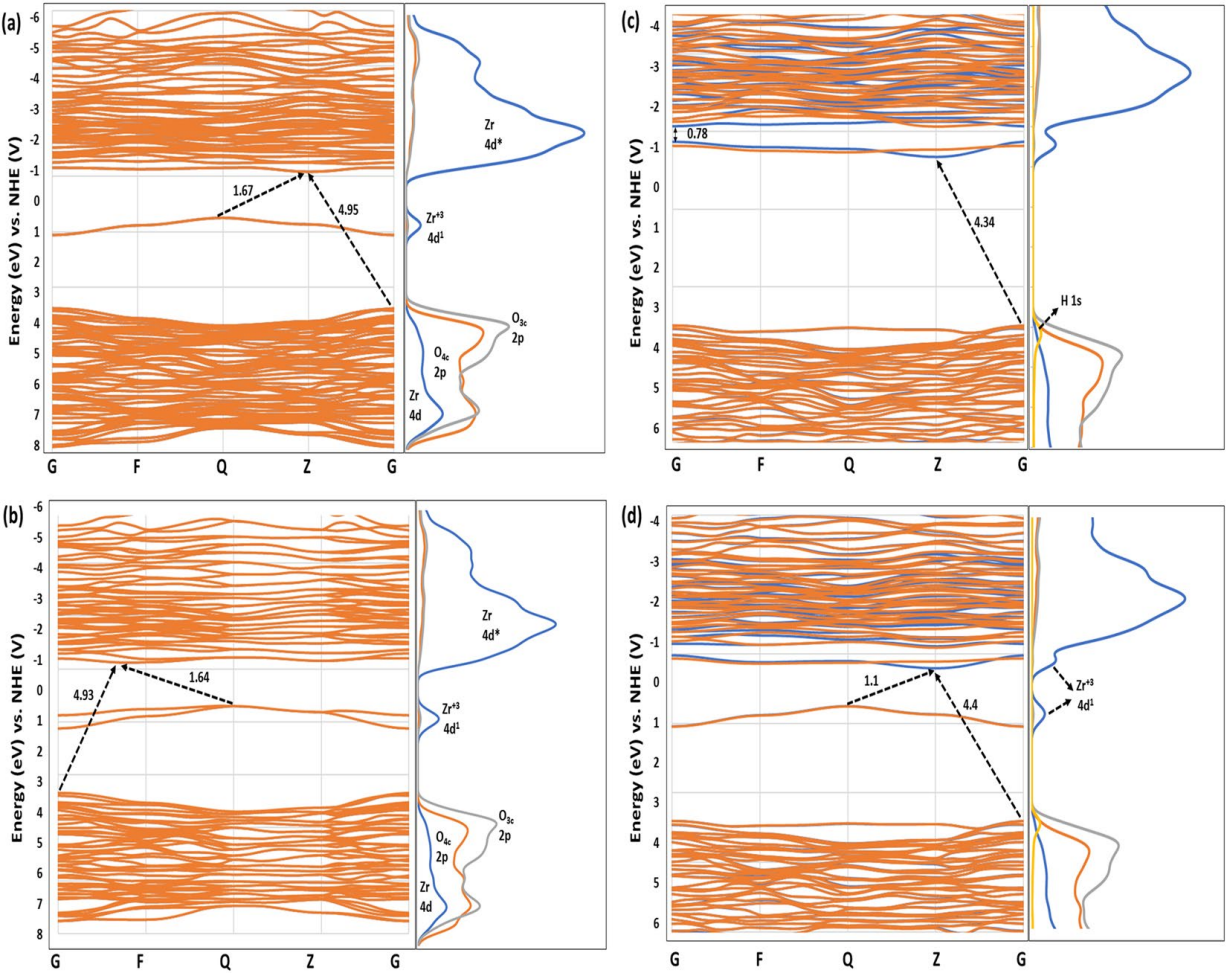
Band structure and PDOS of (**a**) 1O_v_, (**b**) 2O_v_, (**c**) 1Hov, and (**d**) Hov-O.

In photocatalysis applications, the relative band edge position of a semiconductor to the redox potentials of the adsorbate controls the ability of the semiconductor to undergo photoexcited electron transfer to the adsorbate on its surface. When the CB edge potential is more negative than the reduction potential of the adsorbate, the photogenerated electrons would reduce it. When the VB edge potential is more positive than the oxidation potential of the adsorbate, the photogenerated holes would oxidize it. Thus, the possible photocatalytic mechanism can be identified through the study of the electronic band structure. The relative dispositions of the VB and CB potentials of the defected structures with the best optical absorption were calculated according to their absolute electronegativities and with respect to the normal hydrogen electrode (NHE) using the following equations:

3 $${E}_{VB}=\chi -{E}_{e}+\frac{1}{2}{E}_{g}^{optical}$$

4 $${E}_{CB}={E}_{VB}-{E}_{g}^{optical}$$

where χ is the absolute electronegativity of the structure, which is the geometric mean of the electronegativities of the isolated component atoms, E_e_ is the energy of free electrons on the hydrogen scale (approximately 4.5 eV), and E_CB_ and E_VB_ are the CB and VB edge potentials, respectively. The band edge illustration along with the PDOS of pristine and defected structures with respect to the normal hydrogen electrode (NHE) are shown in Fig. 6. The potential of water oxidation (H_2_O/O_2_) was set to 1.23 V and the potential of hydrogen generation (H^+^/H_2_) to 0 V. Localized energy levels in the bandgap of the defected structures (Fig. 2) were also represented. Only structures with good absorption in the visible region are presented. For the H_Ov_-Ov and 1H_Ov_ structures, the CBM is shifted downward to the redox potential of hydrogen generation by 0.3 and 0.39 V, respectively compared to the pristine and oxygen-deficient structures, indicating more favorable photocatalytic activity for hydrogen generation. Note that the VBM is still well below the potential of water oxidation. It is noticed in Fig. 6c,d that hydrogen defects introduced partially filled CBM, which can explain the tail-like Vis-IR absorption. Also, all bandgaps are indirect. Note that while the generation of electron-hole pairs in a direct band gap semiconductor could be easier compared to the indirect counterpart, recombination in direct band gap semiconductors has a much higher probability.

**Figure 7:**
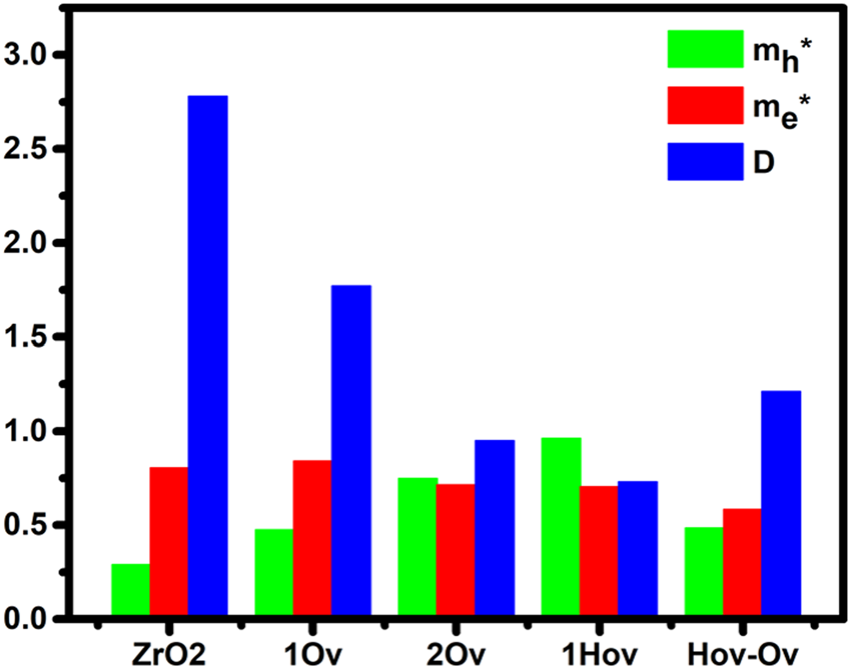
Average effective mass of holes and electrons and its ratio (D). Note: for 1O_v_, 2O_v_, and H_Ov_-O_v_, the ${{\rm{m}}}_{{\rm{h}}}^{\ast }$ is of the bandgap state.

In addition to the band edge positions, photocatalytic activity is closely related to the effective masses of photogenerated carriers and the exciton binding energy, which represents the energy required to ionize an exciton (electron-hole pair) from its lowest energy state. The effective mass of photogenerated charge carriers is related to the band dispersion at the extreme points of the bandgap edges and can be calculated using Eq. 5:

5 $${m}^{\ast }={\hslash }^{2}{(\frac{{d}^{2}{E}_{k}}{d{K}^{2}})}^{-1}$$

where m* is the effective masses of holes (${{\rm{m}}}_{{\rm{h}}}^{\ast }$) and electrons (${{\rm{m}}}_{{\rm{e}}}^{\ast }$) along various directions in unit of m_e_ (the free-electron mass), k is the wave vector, and E_k_ is the energy corresponding to k. Table 4 shows the calculated effective masses and Fig. 7 shows the average effective masses and ${{\rm{m}}}_{{\rm{e}}}^{\ast }$ to ${{\rm{m}}}_{{\rm{h}}}^{\ast }$ ratio for all studied materials. Note that the effective masses of the Hov-O_v_ structure are the lightest as compared to the other structures with high absorption (2O_v_ and 1Hov), indicating faster charge transfer and consequently higher mobility^35^. Smaller effective masses and higher mobility of electrons and holes result in a better performing photocatalyst^36^. Furthermore, to analyze the electron-hole recombination rate, the relative ratio (D) of the electron effective mass to the hole effective mass was calculated using Eq. 6 ^37^. Larger ${{\rm{m}}}_{{\rm{e}}}^{\ast }$ than ${{\rm{m}}}_{{\rm{h}}}^{\ast }$ results in larger D, indicating less recombination of charge carriers and more mobility. Among 2Ov, H_Ov_, and H_Ov_-O_v_ structures with the best optical absorption, the H_Ov_-O_v_ structure showed the highest D and thus the lowest recombination rate, see Fig. 7.

6 $$D=\frac{{m}_{e}^{\ast }}{{m}_{h}^{\ast }}$$

**Table 4:** Calculated effective masses of charge carriers in the studied structures.

Structure		${{\bf{m}}}_{{\bf{h}}}^{\ast }$ (VBM)		${{\bf{m}}}_{{\bf{h}}}^{\ast }$ (bandgap state)		${{\bf{m}}}_{{\bf{e}}}^{\ast }$ (CBM)	
Pure ZrO_2_	Direction	G → F	G → Z	—		G → F	G → Z
	Calculation	0.331	0.249			0.389	1.22
	Average	0.29				0.805	
1O_v_	Direction	G → F	G → Z	Q → F	Q → Z	G → F	
	Calculation	0.334	0.262	0.442	0.508	0.841	
	Average	0.298		0.475			
2O_v_	Direction	G → F	G → Z	Q → F	Q → Z	F → Q	F → G
	Calculation	0.325	0.215	0.986	0.512	0.63	0.797
	Average	0.27		0.749		0.714	
1H_Ov_	Direction	G → F	G → Z	—		Z → G	Z → Q
	Calculation	0.813	1.11			0.413	0.994
	Average	0.962				0.704	
H_Ov_-O_v_	Direction	G → F	G → Z	Q → F	Q → Z	Z → G	Z → Q
	Calculation	0.926	1.34	0.465	0.503	0.415	0.752
	Average	1.133		0.484		0.584

**Figure 8:**
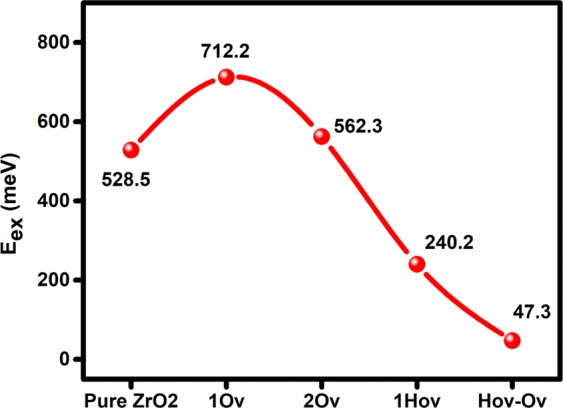
The calculated exciton binding energy of pure and defected m-ZrO_2_ at 0 K.

Also, the exciton binding energy (E_ex_) strongly affects the photocatalytic activity^38^. Generally, for an efficient separation of excitons, the exciton binding energy is required to be lower than K_B_T ~25 meV at room temperature^39^. The exciton binding energy was calculated using Eq. 7.

7 $${E}_{ex}=\frac{{m}^{\ast }{e}^{4}}{2{h}^{2}{\varepsilon }_{r}^{2}}\,$$

where m^*^ is the reduced effective mass of the electron−hole (1/m^*^ = 1/${{\rm{m}}}_{{\rm{e}}}^{\ast }$ + 1/${{\rm{m}}}_{{\rm{h}}}^{\ast }$), e is the elemental charge, h is Planck’s constant, and ε_r_ is a relative permittivity or dielectric constant^40^. As shown in Fig. 8, hydrogen doping significantly decreases the E_ex_, where the lowest energy was observed for the H_ov_-O_v_ structure. However, the obtained E_ex_ of 47.3 meV is still higher than the 25 meV. Note that DFT calculations in this work were performed at 0 K not room temperature. Therefore, the E_ex_ of the H_ov_-O_v_ structure is expected to decrease at the room temperature based on the previous experimental findings that demonstrate the temperature effect on the reduction of E_ex_^41^.

## Conclusion

First-principles calculations using DFT + U approach were carried out to study the effects of hydrogen doping on the electronic structure and optical properties of pristine and oxygen-deficient monoclinic Zirconia. The role of the substantial occupation of hydrogen atoms in oxygen vacancies in tweaking the electronic and optical properties of the monoclinic Zirconia is demonstrated. Different defect structures, sites, and concentrations were investigated to predict the possible structure under different preparation conditions. The results showed that the defect effects are strongly site- and concentration-dependent. At low oxygen vacancy concentration, hydrogen mono-occupying the vacancy was found to increase the dielectric constant and to introduce shallow states below the CBM that can fix the carrier traps of the vacancy without introducing fixed charge centers. Also, the results indicate the possibility of oxygen vacancy to be filled with two hydrogen atoms. At high oxygen vacancy concentration, the presence of hydrogen occupied vacancy along with hydrogen free vacancy was found to extremely increase the optical absorption, the dielectric constant, and the electron-photon interaction, which as a result should improve the charge carrier extraction efficiency. This enhancement is ascribed to the formation of shallow and localized states right below the CBM and the band gap narrowing. From the band edge position of the defected structures along with the electronic and optical properties, hydrogen defects can significantly increase the photocatalytic activity for hydrogen generation by shifting the CBM below to the redox potential of hydrogen generation. This study can provide a detailed guide and description of the role of hydrogen defects in fabricating new optimal materials for photovoltaic and photocatalytic applications.
